# Investigation of a Natural Antibiotic's Properties Effective against Resistant Opportunistic Pathogenic Infections

**DOI:** 10.4014/jmb.2409.09018

**Published:** 2025-03-19

**Authors:** Almagul Khassenova, Yerik Shorabayev, Sirina Zhantlessova, Baiken Baimakhanova, Aisha Sultanova, Makpal Yelubaeva

**Affiliations:** 1 Industrial Microbiology LLP, Almaty 050040, Kazakhstan; 2 Al-Farabi Kazakh National University, Almaty 050040, Kazakhstan; 3 Research and Production Center for Microbiology and Virology LLP, Almaty 050010, Kazakhstan

**Keywords:** Actinomycetes, antibiotics, antibacterial properties, spectrum of activity, opportunistic pathogenic bacteria

## Abstract

The widespread use of antibiotics has led to several negative consequences, including the development of multidrug resistance in microorganisms to previously effective medications. Antibiotic resistance is an increasingly critical issue in both inpatient and outpatient settings. The problem is complicated by the decline in the development of innovative drugs to combat the most dangerous and resistant pathogens. One approach to addressing this challenge is to search for producers of new natural compounds with antibiotic activity. The primary objective of this study was to identify streptomycetes capable of synthesizing complex antibiotics with antibacterial properties. In this study, actinomycetes were isolated from the arid soils in Kazakhstan, followed by the analysis of their antagonistic properties. The greatest interest was generated by isolate No. 312, obtained from rocky soils of the Almaty region and grown on oat agar. The article describes the biochemical, cultural-taxonomic, and antagonistic properties of the new actinomycete. The new antibiotic exhibited the strongest antagonistic activity against clinical strains of *Staphylococcus aureus* (MRSA) and *Escherichia coli* (ESBL) with various types of resistance. The inhibition zone diameter for *S. aureus* was 32 ± 0.2 mm, and for *E. coli*—20 ± 0.1 mm. This field is actively advancing in leading countries worldwide and holds particular importance for Kazakhstan, where the biotechnological industry lacks domestic producers of antibiotics currently used in medical practice, as well as producers of new competitive pharmaceuticals.

## Introduction

As antibiotic use has expanded, the number of pathogenic microorganisms exhibiting increased resistance to one or more antibiotics has also risen. This phenomenon has led to a decrease, and in some cases, a loss of effectiveness of the administered drugs. In clinical practice, in addition to multiple drug resistance (MDR) to several types of antibiotics, there is also extensively drug resistance (XDR) to a wide range of antibiotics and pandrug resistance (PDR) to all classes of antibiotics [1-5]. There is a strong correlation between infections caused by antibiotic-resistant bacteria and increased disability and mortality rates [6]. In 2017, WHO developed a list of priority pathogens resistant to antibiotics, designed to serve as a guideline for the development of new antimicrobial drugs [7]. *Acinetobacter* is the causative agent of severe pneumonia, skin wound infections, urinary tract infections, and sepsis [8, 9]. Some types of drug-resistant strains of *Pseudomonas aeruginosa* are resistant to almost all antibiotics, including carbapenems [10]. *Enterobacteriaceae* produce broad-spectrum β-lactamases that degrade common first-line antibiotics, rendering them ineffective against a wide range of bacteria. Polyresistant strains of *Klebsiella pneumoniae* and *Escherichia coli* produce extended-spectrum beta-lactamases [11]. About 30% of all enterococcal infections are resistant to vancomycin, which significantly reduces the number of treatment options [12]. Typhoid fever requires antibiotic treatment, which is complicated by the increased resistance of strains. Progress in the prevention of sepsis caused by methicillin-resistant strains of *Staphylococcus aureus* (MRSA) [13, 14] is steadily slowing down. Four first-line antibiotics are recommended for the treatment of tuberculosis, however, the development of resistance to any of these drugs limits treatment options and exposes the patient to the risk of incurable tuberculosis [15].

According to WHO, in 2019 there were only 32 drugs under development that were aimed at combating the most dangerous pathogens, and only 6 of them were recognized as innovative [16]. Despite significant progress in the field of chemical synthesis and engineering biosynthesis of antimicrobial compounds, the need for antibiotics has not yet been met. Therefore, actinomycetes remain the most versatile producers of new metabolites with antimicrobial action [17-19]. The structural diversity of natural products provides a broad scope of work for the discovery of new compounds with important applications in chemistry, biology, and medicine, as they have fewer adverse secondary effects compared to synthetic compounds [20, 21]. Most studies on actinomycetes focus on the genus *Streptomyces*, therefore, the majority of isolated compounds are produced by *Streptomyces* sp.

Among the secondary metabolites of the deep-sea culture *Nocardiopsis alba*, a family of new diketopiperazine antibiotics with pronounced antitumor activity has been discovered [22]. The ongoing search for new natural antibiotics remains important, as demonstrated by numerous studies focused on isolating novel antibiotics from a variety of natural sources [22-24].

Additionally, new antibiotics, androprostamines A and B, isolated from *Streptomyces* sp. MK 932-CF8, exhibit low toxicity and effectively suppress the androgen receptor, a key target in prostate cancer [23]. Omadacycline, a newly developed aminomethylcycline antibiotic, was obtained for both intravenous and oral administration. Furthermore, a next-generation aminoglycoside antibiotic, plazomicin, has been isolated; it is effective against both gram-positive and gram-negative bacteria and demonstrates resistance to the most clinically significant aminoglycoside-modifying enzymes [24]. Other newly discovered antibiotics include penibacterin, a broad-spectrum lipopeptide antibiotic [25]; battacin, a cyclic lipopeptide antibiotic with a potent bactericidal effect against gram-negative bacteria, capable of actively disrupting their membranes [26]; and mangromycins A and B, which exhibit notable antitrypanosomal activity [27].

A new class of boron-containing antibacterial drugs has been discovered [28], and the "old" antibiotic nibomycin has been rediscovered, now exhibiting new properties, including efficacy against quinolone-resistant strains of *S. aureus* [29].

Teixobactin, a novel antibiotic identified in 2015, shows promise for the treatment of bacterial lung diseases caused by multidrug-resistant pathogens, tuberculosis, and bacterial complications associated with COVID-19 [30]. It is a cyclic depsipeptide containing an unusual amino acid enduracididine. It is the first new class of antibiotic which acts on unique targets in cell wall synthesis pathway. It binds to a highly conserved non-peptide motif of peptidoglycan precursor (lipid II) and teichoic acid precursor (lipid III), resulting in inhibition of cell wall synthesis and subsequent lysis. It has shown excellent activity against a wide range of gram positive bacteria, including multidrug resistant organisms such as methicillin-resistant *Staphylococcus aureus* (MRSA), vancomycin-intermediate *S. aureus* (VISA), vancomycin-resistant enterococci (VRE), *Clostridium difficile*, *Streptococcus pneumoniae*, and *Mycobacterium tuberculosis* [31].

Another innovative pleuromutilin antibiotic, lefamulin, received FDA approval in 2019 [32]. Lefamulin is used to treat community-acquired bacterial pneumonia, including infections caused by drug-resistant *Streptococcus pneumoniae*. It functions by inhibiting bacterial protein synthesis through binding to the peptidyl transferase center of the bacterial 50S ribosome, thereby preventing the binding of transfer RNA to the peptide. This mechanism provides an alternative treatment option in cases where resistance to older antibiotics has developed [33, 34].

Thus, the study of the antimicrobial and physicochemical properties of new natural antibiotics is a necessary step to identify the prospects of their further research, namely, the selection of drugs with high antibacterial or antifungal activity, potentially valuable for medicine, to study their chemotherapeutic properties and conduct preclinical trials.

## Materials and Methods

### Isolation of Actinomycetes

The search for antibiotic producers was conducted among 378 isolates of actinomycetes, isolated from various soils of the Almaty region. The isolation of actinomycetes was carried out using the generally accepted method of culturing on the surface of starch-ammonia agar (SAA). The actinomycete was cultured on oatmeal agar and Gause No. 2 medium. The antibacterial properties of the actinomycete were assessed using agar plugs and well diffusion methods [35]. Strains of gram-positive (*S. aureus*, *Salmonella abony*) and gram-negative (*K. pneumoniae*, *E. coli*) opportunistic bacteria were employed as test microorganisms.

### Determination of Antibacterial Activity

Antibacterial activity was determined using the agar diffusion method (agar plug) on nutrient agar (Hi-Media). Antimicrobial and antifungal activity is increased by diffusion in agar on nutrient agar for bacterial and yeast-like test microorganisms; for mold fungi, Czapek-Dox agar is used [35]. The inoculum was prepared by directly suspending colonies in a sterile isotonic solution to a density of 0.5 according to the McFarland turbidity standard.

To assess antibiotic activity in Petri dishes seeded with test cultures in deep growth (CFU 10^6^/ml), wells were made using a standard drill (d = 7 mm), and then the filtrate of the native solution or biomass extract in an amount of 0.1 ml was added to the wells using a Pasteur pipette. Sterile clean media and ethanol were used as controls.

The diameter of the growth inhibition zones of bacterial test microorganisms was measured after incubation at a temperature of 37°C for 24 h, and of filamentous and yeast-like fungi at a temperature of 25°C for 72 h.

To evaluate the antagonistic properties, agar plugs of actinomycete cultures were prepared using a standard drill (diameter = 7 mm). These plugs were aseptically deposited on the agar plates inoculated with submerged cultures of test microorganisms (CFU 10^6^/ml). Sterile pure nutrient media served as the control. Then, agar plates were incubated at 37°C for 24 h and the antimicrobial activity was detected by measuring the diameter of inhibition zones of test microorganisms around the agar plug.

The selection of clinical strains with different types of resistance was carried out at the JSC “Central Clinical Hospital”, Almaty. Identification of clinical strains of opportunistic pathogens and determination of their resistance to drugs was performed on an automatic bacteriological analyzer ("MINI API", BIO MERIEUX). The laboratory follows EUCAST recommendations.

The antimicrobial activity of antibiotics was studied by diffusion in agar on nutrient agar. The diameter of the inhibition zones of test microorganisms around agar plugs was measured after 24 h incubation (37°C).

### Determination of Taxonomic Position and Molecular Genetic Identification

Taxonomic analysis of the actinomycete strain was performed following the method described by Shirling and Gottlieb [36]. The type of spore chains was determined in a mature culture on the 10^th^ day of growth. The morphological observations were examined using a Leica DMLS trinocular microscope equipped with a Leica DC 300F digital video camera. The spore surface was analyzed using a Jem-100B transmission electron microscope without sample fixation.

Cultural characteristics, including the colour of the aerial and substrate mycelium and soluble pigments, were conducted on the 14^th^ day of culture growth using the ISP 3-7 diagnostic media recommended by Shirling and Gottlieb [36] and Gause *et al*. [37]. Colour identification was determined according to the Bondartsev colour scale [38]. Utilization of carbohydrates by actinomycete strains was assessed with Pridham and Gottlieb carbon nutrient medium using the method recommended by Shirling and Gottlieb [36]. The physiological and biochemical characteristics of the strain were examined using generally accepted methods.

To obtain a spore material, the actinomycete strain was cultured at 28°C for 10 days on agar Gauze’s No.1 medium or oatmeal agar. Inoculation of the liquid nutrient medium was conducted by introducing an inoculum of actinomycete spores at a concentration of 10^9^ (1 ml of inoculum: 100 ml of media).

Genetic determination of actinomycete strain was performed by culturing them on mineral agar Gause medium No.1. Next, the genomic DNA was extracted using the PureLink Genomic DNA Kit (Invitrogen, USA) according to the manufacturer's instructions. The concentration of the obtained DNA was measured using a Qubit 2.0 fluorometer. Sequencing of DNA genetic libraries on the MiSeq system was prepared following the 16S Metagenomic Sequencing Library Preparation protocol [39]. The 16S rRNA gene was amplified using primers to V3 and V4 regions and oligonucleotide adaptors Illumina. The analysis of the DNA sequences of the studied strains was performed on the MiSeq instrument following the MiSeq^®^ System User Guide. Bioinformatic analysis of the data obtained from actinomycete sequencing was conducted using the MiSeq Reporter software.

### Biosynthesis and Release of Antibiotics

Biosynthesis of biologically active substances was carried out in 750 ml Erlenmeyer flasks containing 100 ml of medium and incubated on a circular shaker at 180-200 rpm and 28°C for 120 h. The pH was measured using MP 220 Mettler Toledo AB 54-S pH meter. The mycelium was collected from a culture medium by centrifugation (2,000 ×*g*, 20 min) or filtration. The biomass was then pressed to remove residual moisture, reaching 75% moisture content, weighed, and extracted with acetone at a 1:3 ratio. The acetone extracts were separated by filtration.

Antibiotics were isolated from both the biomass and the native solution of the producer strain using the following extraction methods: extraction with n-butanol and ethyl acetate (3:1) from the culture liquid, and with acetone (1:3) from the mycelium.

The extracts were concentrated under vacuum using an IKA RV 10 basic rotary evaporator. After acetone removal, the aqueous residue was extracted with n-butanol or ethyl acetate. The extracts were re-evaporated under vacuum to obtain a dry residue, which was then dissolved in 50% ethanol.

### Physicochemical Properties of the Obtained Antibiotics

The group identification of antibiotic A-312 was conducted by examining its biological and physicochemical properties through various methodologies, including determination of the antimicrobial spectrum, thin-layer chromatography (TLC), spectrophotometry, and infrared (IR) spectroscopy.

Antibiotic A-312 from the culture medium and biomass of 312 strain were chromatographed on Sorbfil (Sorbpolymer, Russia) and DC-Alufolien Kieselgel 60 (Merck, Germany) plates. Optimal chromatographic conditions for the antibiotic complex were determined using various solvent systems: n-butanol-acetic acid-ethanol (1:1:4), n-butanol-ethanol-water (1:4:1), hexane-methanol-chloroform (3:1:2), chloroform-methanol (7:1), hexane-methanol:ethanol (3:1:1). The antibiotics were detected visually, through UV light luminescence using a chromatoscope (UVS), and via the bioautographic method with *S. aureus* as the test organism.

The absorption spectra of the complex antibiotic and its components in the UV and visible regions were measured in 96% ethanol using a Cary 60 UV-Vis spectrophotometer (Agilent Technologies). IR spectra were recorded on a Nicolet 5700 spectrometer in tablets with KBr.

### Statistical Analysis

All studies were conducted in three to five replicates. All data were subjected to statistical analysis, which was carried out using the “Statistica 10.0” software package [38]. Statistical analysis was performed by calculating the means and standard deviations of the results.

## Results

### Selection of Actinomycetes

A total of 378 isolates of actinomycetes from soils of the Almaty region were isolated into the pure cultures. The predominant species of actinomycetes are *Coerulescens*, *Albus*, *Flavus*, *Aureus*, *Chromogenes*, *Ruber*. A collection of soil actinomycetes has been created. 183 isolates exhibited antibacterial activity, and 110 isolates exhibited antifungal activity. 31 strains had high activity (more than 20 mm) against *S. aureus*, 15 soil actinomycetes were the most active against *E. coli* (Table 1).

**Fig. 1:**
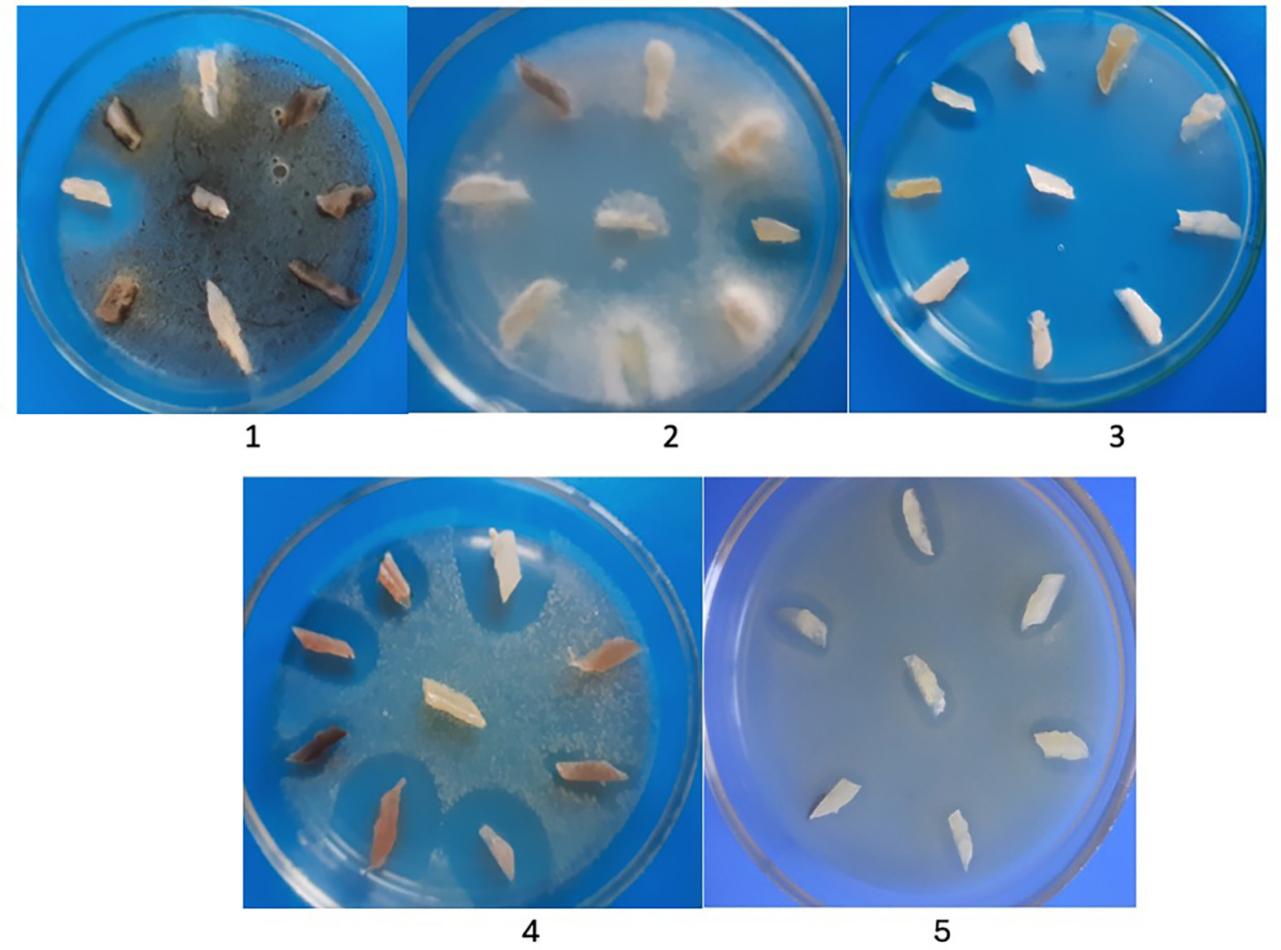
Antagonistic properties of actinomycetes against fungi (1 - *Aspergillus niger*, 2 - *Fusarium solani*, 3 - *Candida albicans*); gram-positive (4 - *Staphylococcus aureus*) and gram-negative bacteria (5 - *Escherichia coli*).

The antifungal activity of the isolated cultures against yeast-like and filamentous fungi was determined. 110 strains showed activity against filamentous and 48 against yeast-like fungi. 45 strains had activity from 20-25 mm to *A. niger*, and 21 strains to *F. solani*. Thirteen strains showed high activity against *C. albicans* and *C. utilis* (Fig. 1).

Table 1 shows data on isolates exhibiting antibacterial activity. From among them, actinomycetes with high activity against gram-positive and gram-negative microorganisms were selected for subsequent work.

The table includes isolates that showed high activity against *S. aureus*. Analysis of our results showed that 76 (20.1%) actinomycete isolates showed antibacterial properties against the studied gram-positive and 30 (7.9%) isolates against gram-negative test microorganisms. The isolates showed the highest activity against *S. aureus* 208, 211, 226, 229, 312, 324, 327, 330, 333, 342, 358 (diameter of the growth inhibition zone - 30.1 ± 0.2 mm and above), against *E. coli* - isolates No. 208, 211, 226, 229, 312, 324, 330, 342 (diameter of the growth inhibition zone -20.5 ± 0.1 mm and above). 13 isolates (12.3%) of actinomycetes with antibacterial properties had a wide spectrum of antibacterial action and were active simultaneously against gram-positive and gram-negative bacteria.

The new isolate 312 exhibited the highest activity against all the studied test-organism strains. The strain is characterized by its pigment-forming ability and production of a broad-spectrum antibiotic.

### Determination of Taxonomic Position and Implementation of Molecular Genetic Identification

**Fig. 2:**
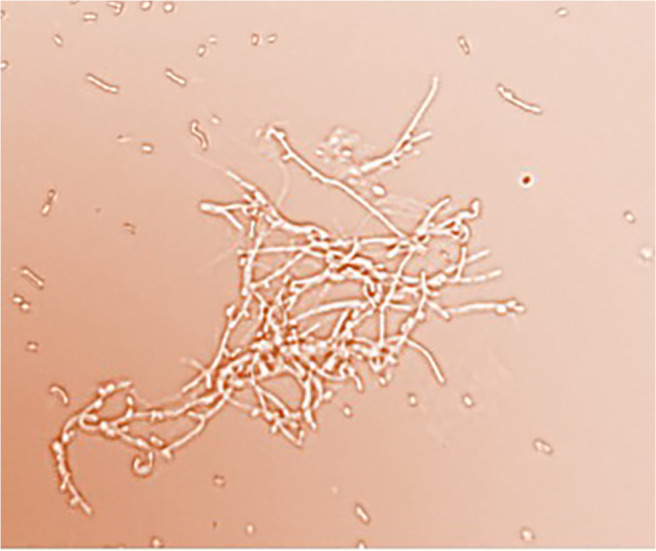
Mycelium of actinomycete 312 (magnification 18x100).

**Fig. 3:**
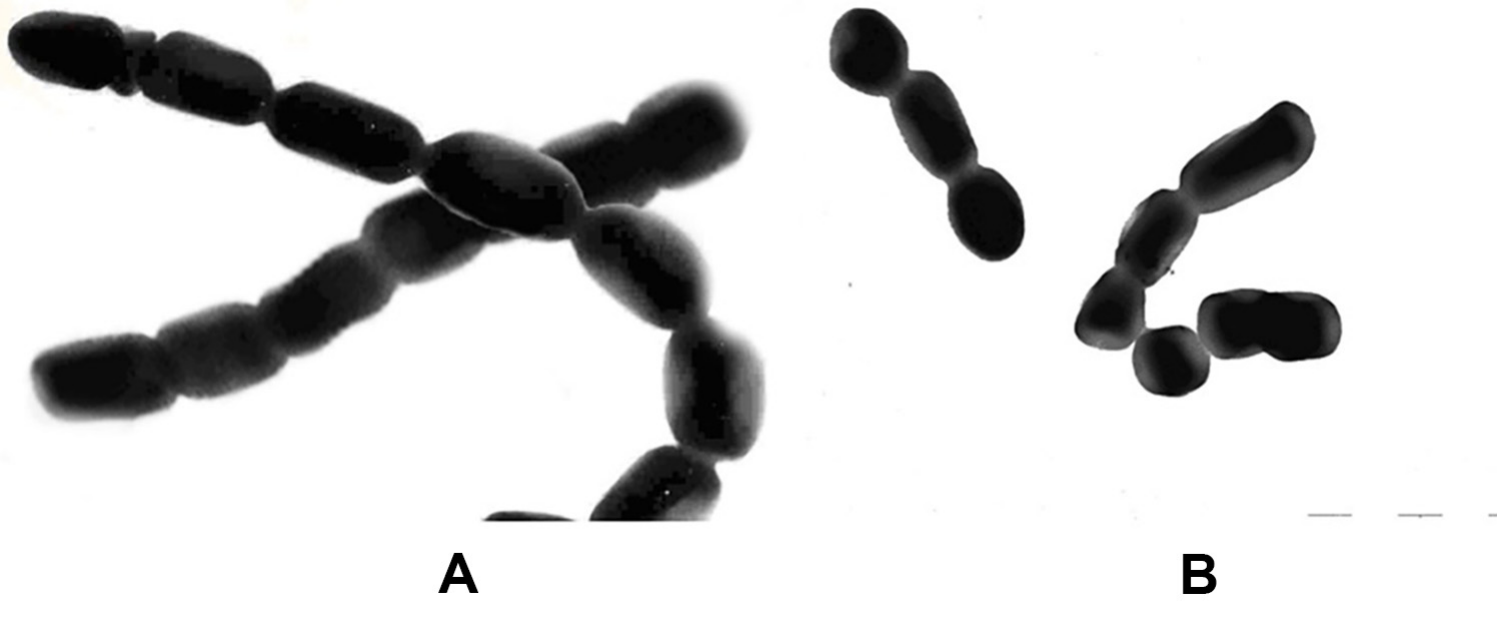
Shape and surface of spores of strain 312 (magnification x 20000).

The cultural, taxonomic, and biochemical properties of the new isolate have been studied. Strain 312 is characterized by long, straight, branching aerial mycelial hyphae, with chains containing more than 10 spores. Based on its sporulation pattern, strain 312 is classified as the RF type, featuring straight or twisted spore chains and producing oval-shaped spores with a smooth surface (Figs. 2 and 3).

Culturing on diagnostic media demonstrated that strain 312 forms a pigment ranging from pink (glucose-aspartic), and brown (glycerol-aspartic) to burgundy (oatmeal agar) and purple on other media.

**Fig. 4:**
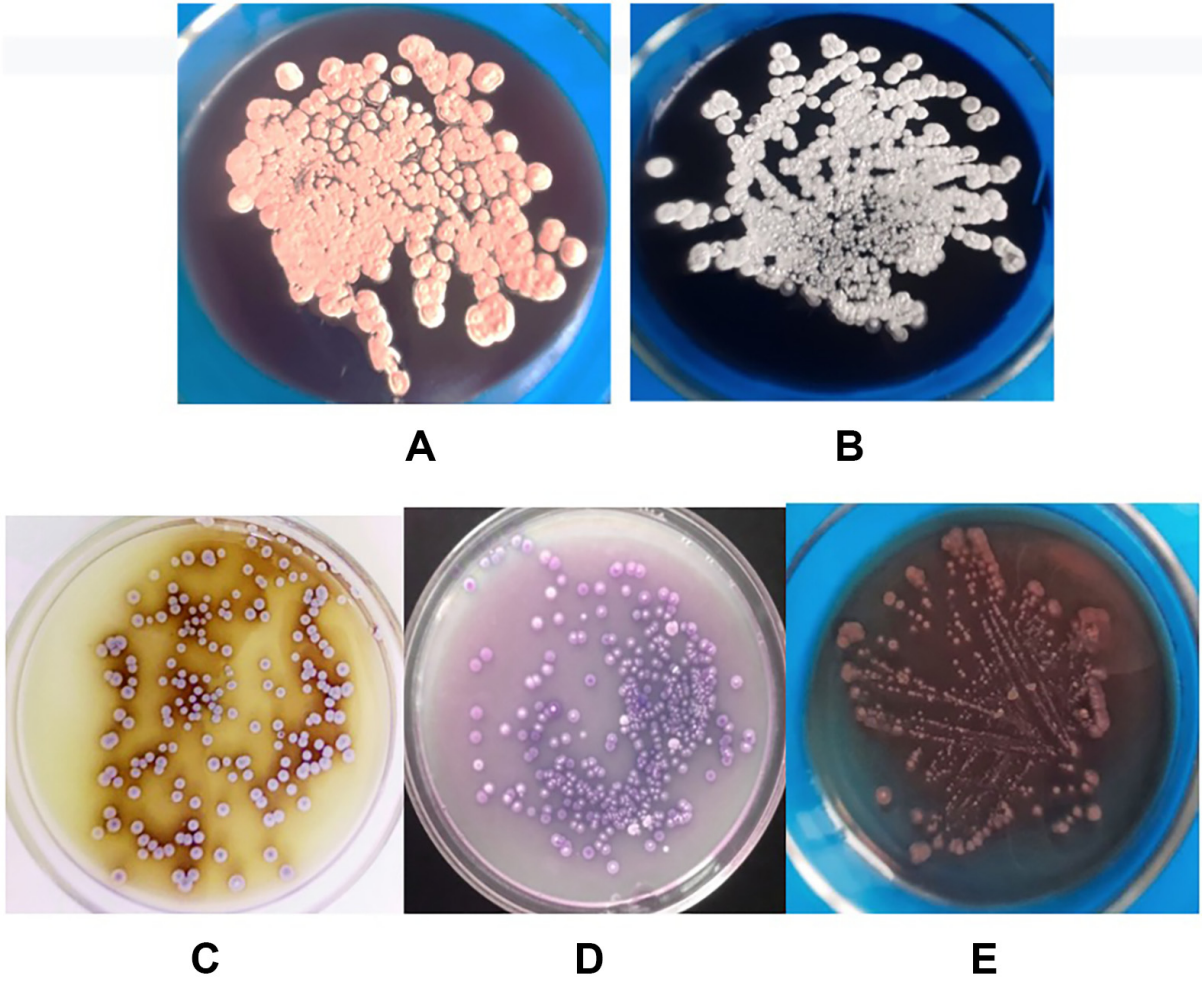
Growth of strain 312 on different media ((A) on oatmeal agar, (B) on Czapek’s sucrose-yeast agar, (C) on peptone-yeast agar, (D) on starch-ammonium agar, (E) on glycerol-aspartic agar).

Strain 312 demonstrates abundant growth on oatmeal agar and thrives on organic media such as Gauze No. 2, Prauser 79, and sucrose-yeast Czapek agar. Moderate growth is observed on Gauze media No. 1, sucrose-nitrate Czapek agar, starch-ammonia agar, and peptone-yeast agar. In contrast, growth is weaker on glucose-aspartic, glycerol-aspartic, and glucose-nitrate media. The aerial mycelium of strain 312 varies in color across diagnostic media, ranging from white to light pink, while the substrate mycelium shifts from light purple to dark purple and brown. Additionally, strain 312 produces a melanoid pigment on peptone-yeast agar supplemented with iron (Table 2, Fig. 4).

Strain 312 efficiently utilizes most of the studied carbon sources. It was found to metabolize glucose, sucrose, fructose, arabinose, raffinose, and mannitol to a greater extent, while it weakly utilizes xylose, rhamnose, maltose, lactose, and galactose, and does not utilize dulcitol, sorbitol, or inositol. Strain 312 exhibits cellulolytic, gelatinase, and tyrosinase activities.

By the fourth day, it liquefies the upper layer of the gelatin column. It does not show lecithinase or amylolytic activity, lacks denitrifying ability, does not reduce nitrates to nitrites, and does not peptonize milk.

The culture was grown in Petri dishes in an incubator at a temperature of 28°C for 5 days. On oat agar, it forms round colonies with a diameter of 3-5 mm, featuring pale pink aerial mycelium and burgundy substrate mycelium.

**Fig. 5:**
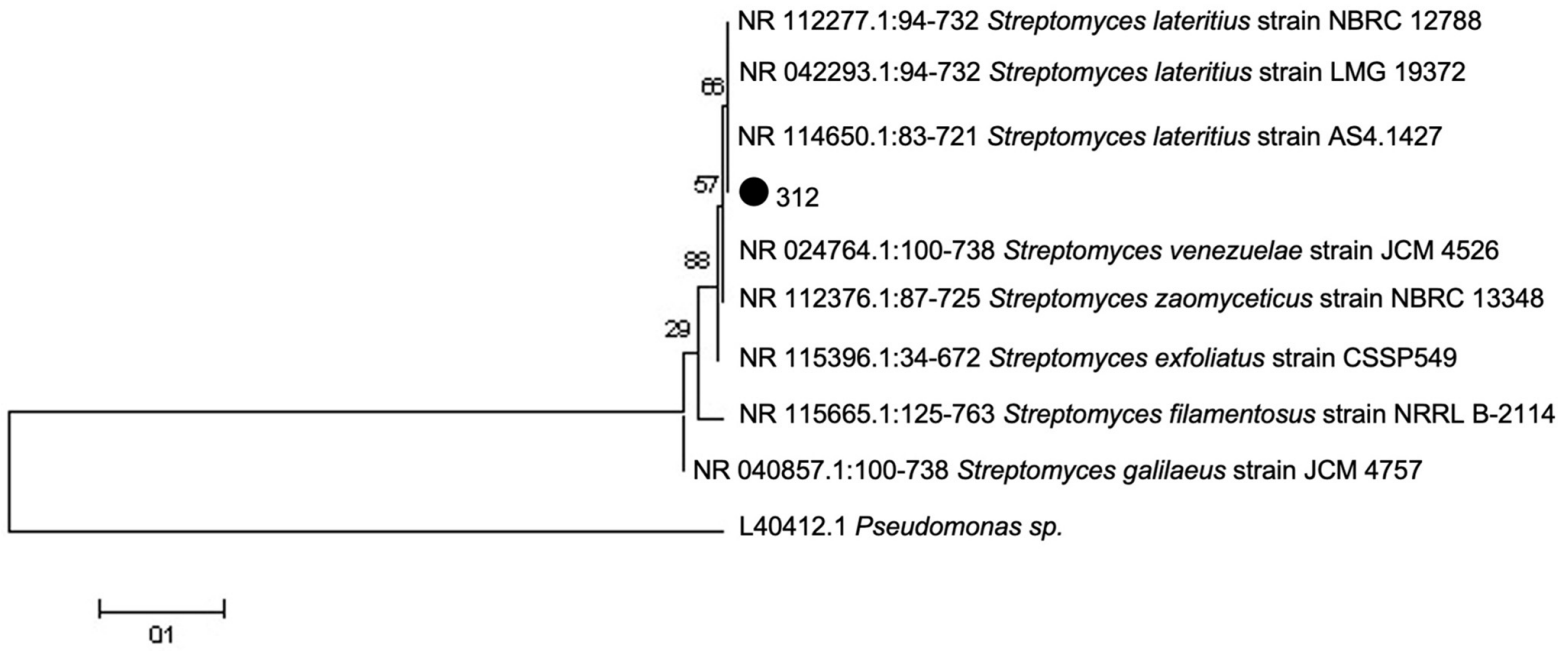
Phylogenetic tree constructed using the neighbor-joining method, demonstrating their closely related strains based on partial 16S rRNA gene sequences. The degree of homology with the closest strain NR 153666.1:56-759 *Streptomyces lacrimifluminis* strain Z1027 was 99.57%.

Based on cultural and morphological characteristics, strain 312 was classified as belonging to the genus *Streptomyces*, specifically within the *Roseoviolaceus* series. Phylogenetic analysis of the 16S rRNA gene sequences of strain No. 312, compared with data from the Greengenes International Database, confirmed that it is a member of *Streptomycetaceae* family, genus *Streptomyces*, species *lateritius*. The strain demonstrated a 99.84% degree of homology with the closest strain, *S. lateritius* AS4.1427 (Fig. 5).

The nucleotide sequence of the 16S rRNA gene is as follows: ATCTGCCCTTCACTCTGGGACAAGCCCTGGAAACGGGGTCTAATACCGGATAACACCGGCTTCCGCATGGAAGCTGGTTGAAAGCTCCGGCGGTGAAGGATGAGCCCGCGGCCTATCAGCTTGTTGGTGGGGTAATGGCCCACCAAGGCGACGACGGGTAGCCGGCCTGAGAGGGCGACCGGCCACACTGGGACTGAGACACGGCCCAGACTCCTACGGGAGGCAGCAGTGGGGAATATTGCACAATGGGCGAAAGCCTGATGCAGCGACGCCGCGTGAGGGATGACGGCCTTCGGGTTGTAAACCTCTTTCAGCAGGGAAGAAGCGAAAGTGACGGTACCTGCAGAAGAAGCGCCGGCTAACTACGTGCCAGCAGCCGCGGTAATACGTAGGGCGCAAGCGTTGTCCGGAATTATTGGGCGTAAAGAGCTCGTAGGCGGCTTGTCACGTCGGGTGTGAAAGCCCGGGGCTTAACCCCGGGTCTGCATCCGATACGGGCAGGCTAGAGTGTGGTAGGGGAGATCGGAATTCCTGGTGTAGCGGTGAAATGCGCAGATATCACGAGGAACACCGGTGGCGAAGGCGGATCTCTGGGCCATTACTGACGCTGAGGAGCGAAAGCGTGGGGAGCGAACAGGA

### Biosynthesis, Isolation, and Antibiotic Activity of the Obtained Substances

**Fig. 6:**
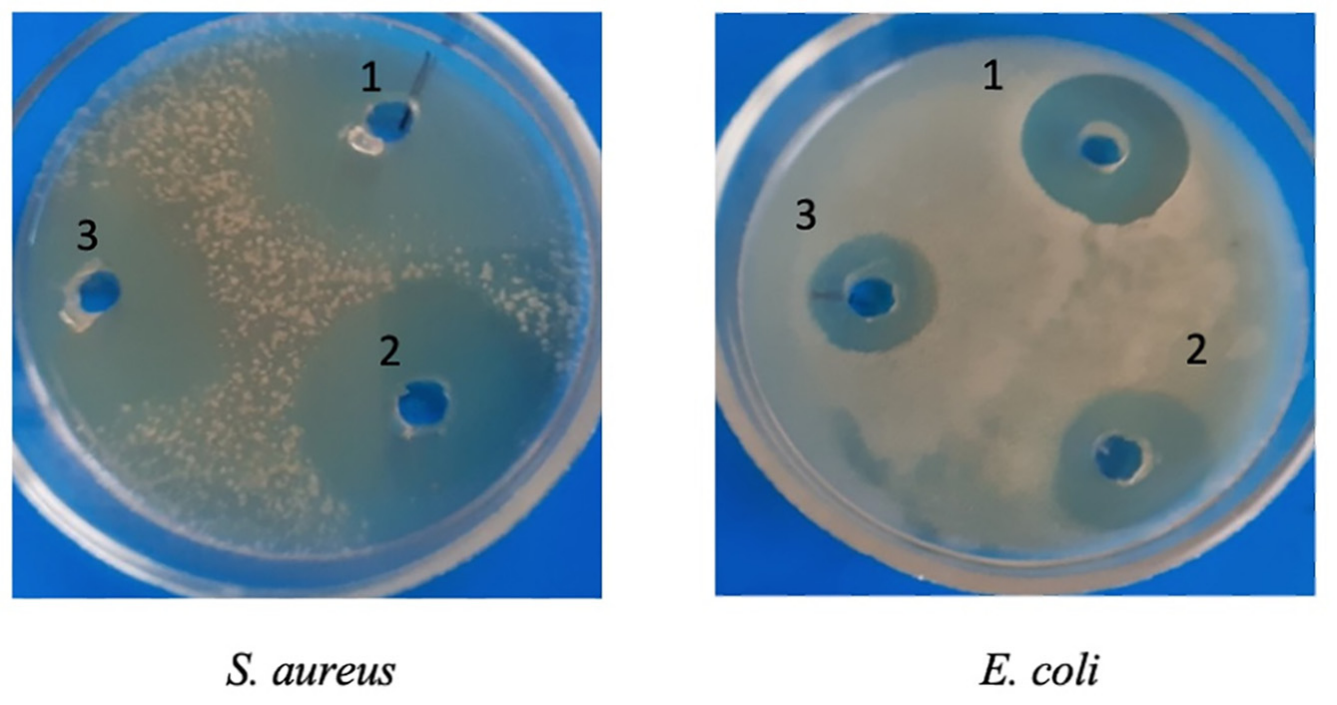
Antagonistic properties of antibiotic A-312 against *S. aureus* and *E. coli* (extracts from culture liquid: 1—ethyl acetate; 2—butanol; extract from mycelium: 3—ethanol).

During deep fermentation of the strain, the antibiotic A-312 accumulates both in the biomass and in the culture liquid. The antibiotics were separately isolated from the biomass and the native solution of the producer strain using the following extraction methods: from the culture liquid by extraction with n-butanol and ethyl acetate, and from the mycelium with acetone. Strain 312 produces an antibiotic pigment that colors the butanol extract blue and the ethyl acetate extract red. The acetone extract from the mycelium has a faint purple color. The antibiotic activity of these extracts against gram-positive and gram-negative bacteria was evaluated using the well diffusion method (Table 3, Fig. 6).

Table 3 shows that the pigment with antibiotic properties is most efficiently extracted with ethyl acetate and exhibits higher antibiotic activity.

The extracts were concentrated under vacuum using a rotary evaporator, yielding the antibiotic in the form of ethanol preparations: A-312-1 from the culture liquid and A-312-2 from the biomass.

The antibiotic activity of A-312-1 was evaluated against clinical strains of opportunistic pathogens with drug resistance. Clinical strains of opportunistic pathogens with various types of resistance were selected to determine the activity of antibiotic A-312 (Table 4).

The clinical strains used as test microorganisms exhibit a high level of resistance to the main groups of medical antibiotics. The antibiotic activity of the obtained antibiotics was determined using the well diffusion method. The data are presented in Table 5.

From Table 5, one can conclude that the concentration of the extracted pigment was the major factor influencing the inhibition activity against various types of microorganisms. The diameter of the growth inhibition zone for clinical strains by antibiotic A 312-1 ranged from 32 mm (*S. aureus* 228) to 15 mm (*E. coli* 446), while for antibiotic A 312-2, it ranged from 25 mm (*S. aureus* 228) to 10 mm (*E. coli* 446).

### Study of the Chemical Parameters of the Obtained Antibiotic Substances

Preliminary identification of antibiotic A-312, produced by the *S. lateritius* 312 strain, was conducted. Chromatographic analysis was performed using Sorbfil (Sorbpolymer, Russia) and DC-Alufolien Kieselgel 60 (Merck) plates with various solvent systems: n-butanol-acetic acid-water (4:1:5), n-butanol-acetic acid-ethanol (1:1:4), n-butanol-ethanol-water (1:4:1), hexane-methanol-chloroform (3:1:2), chloroform-methanol (7:1), and hexane-methanol-ethanol (3:1:1). The results indicated that antibiotic A-312 is a complex preparation comprising at least two components. Thin-layer chromatography data confirmed the identical composition of the components in the preparations obtained from the culture liquid (A-312-1) and from the biomass (A-312-2). The most effective separation of components from the ethyl acetate extract was achieved using the hexane-methanol-chloroform (3:1:2) system, revealing five individual chemical compounds in the ethyl acetate extract and three compounds in the ethanol extract, all of which exhibited luminescence under UV light (Table 6).

**Fig. 7:**
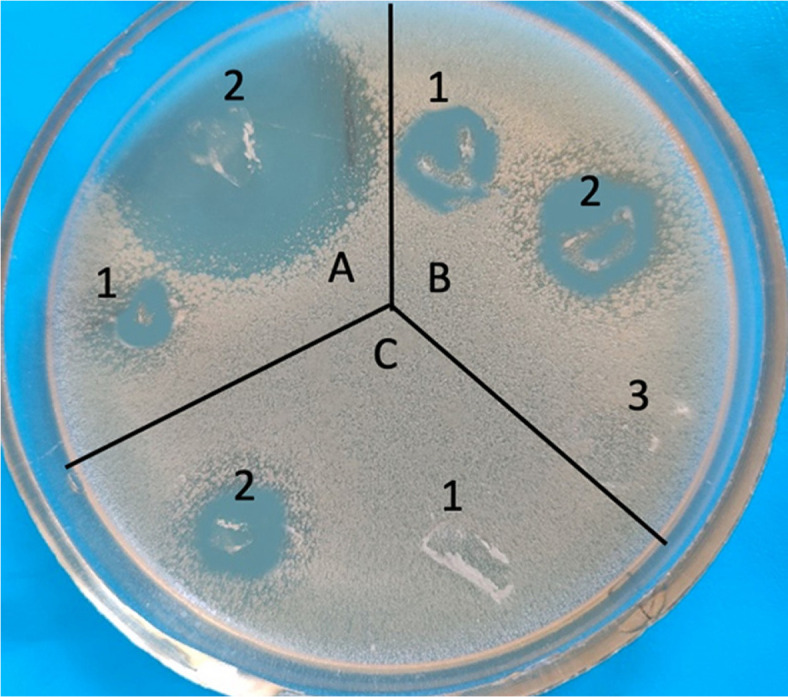
Bioautogram of antibiotic A-312 components in the hexane-methanol (3:1:1) system. Component I (Rf 0.75) and component II (Rf 0.58) exhibit zones of inhibition against *S. aureus*. (**A**) Ethyl acetate. (**B**) Butanol. (**C**) Mycelium.

Using the bioautography method with *S. aureus* as the test organism, it was determined that two components, designated as I and II, exhibit biological activity. These components have Rf values of 0.75 and 0.58 in the hexane-methanol (3:1:1) system (antibiotic A-312-1), and Rf values of 0.94 and 0.89 in the n-butanol-acetic acid-water (4:1:5) system respectively (Fig. 7).

**Fig. 8:**
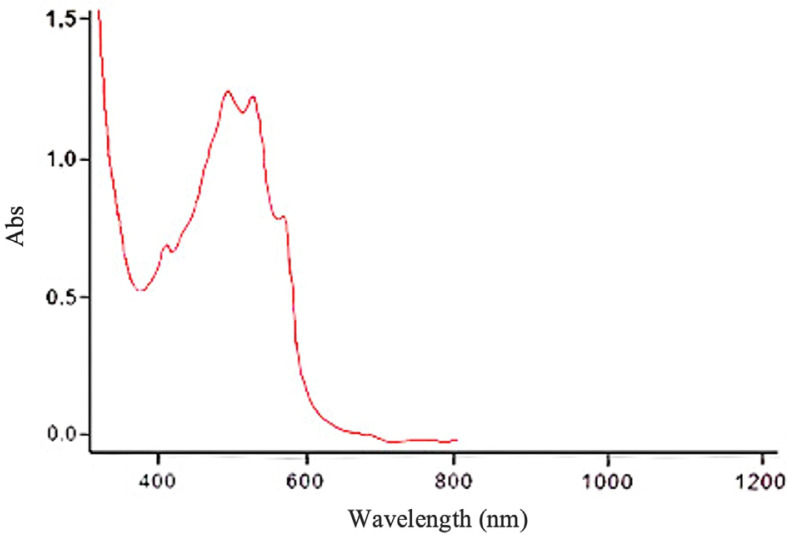
The absorption spectrum of antibiotic A-312 in ethanol.

In the hexane-methanol (3:1:1) system, component II which exhibited significant biological activity, was identified as a homogeneous substance in the thin layer chromatography. The active components were isolated using antibiotic A-312-1 in the zone corresponding to Rf = 0.58 and Rf = 0.75, both showing bright blue fluorescence under UV light. The absorption spectra of the complex antibiotic A-312 and component II are identical, with primary maxima in the visible region at 565, 524, 491, and 409 nm (Fig. 8).

In the hexane-methanol (3:1:1) system, component II which exhibited significant biological activity, was identified as a homogeneous substance in the thin layer chromatography. The active components were isolated using antibiotic A-312-1 in the zone corresponding to Rf = 0.58 and Rf = 0.75, both showing bright blue fluorescence under UV light. The absorption spectra of the complex antibiotic A-312 and component II are identical, with primary maxima in the visible region at 565, 524, 491, and 409 nm (Fig. 8).

**Fig. 9:**
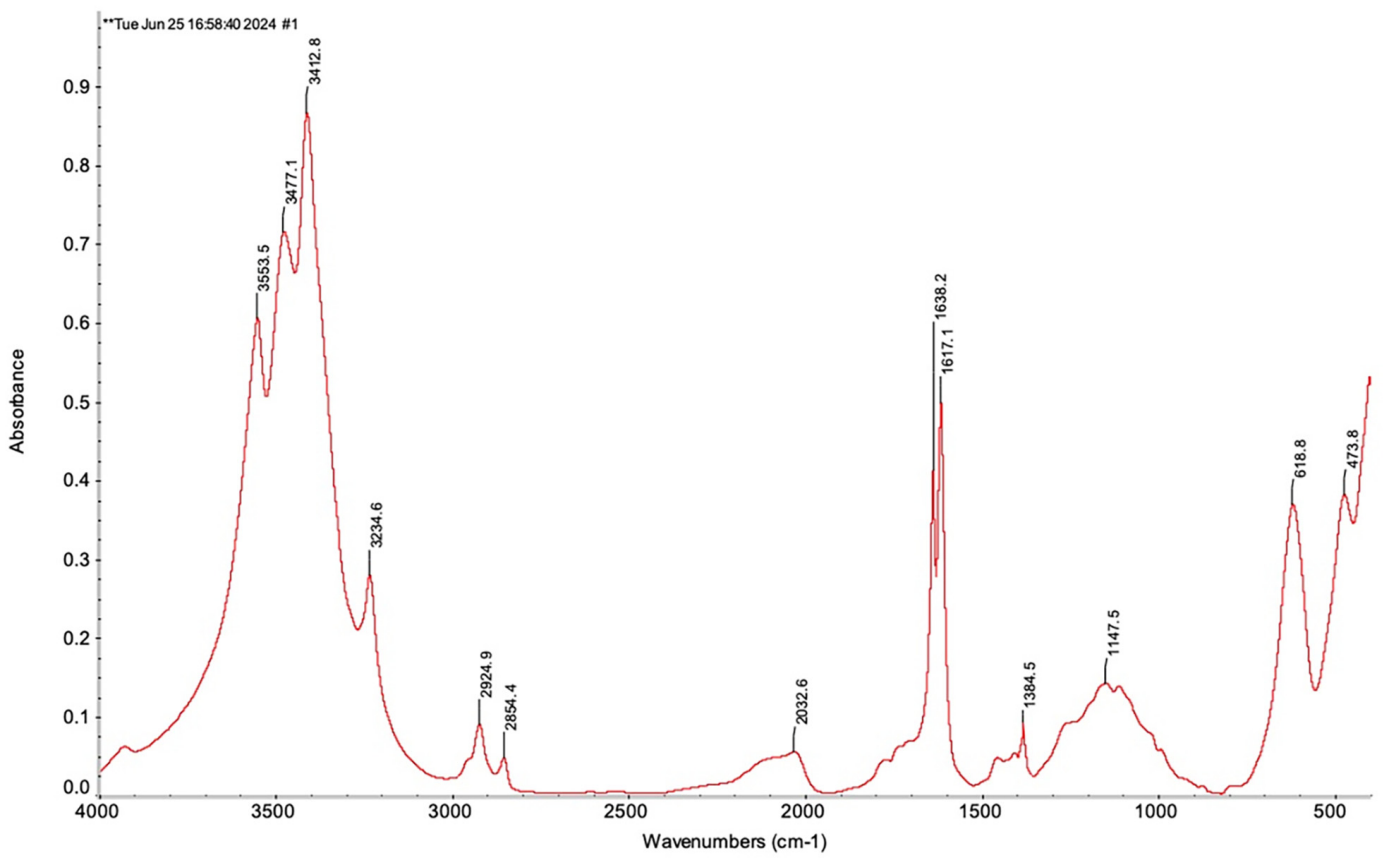
IR absorption spectrum of antibiotic A-312 with KBr.

In the IR spectrum (KBr) there are bands of strong intensity at 3554, 3477, and 3413 cm^-1^ indicating the presence of OH groups in the studied molecule (Fig. 9). However, the double peak observed at 3477 and 3413 cm^-1^ may also suggest the presence of an NH_2_ group.

A narrow, medium-intensity band at 3235 cm^-1^ likely indicates the presence of CH group with terminal acetylene hydrogen, further supported by a broad, low-intensity peak at 2032 cm^-1^, which is a characteristic of an asymmetric C CH bond, possibly conjugated with a C=O carbonyl group.

Low-intensity bands at 2925 and 2854 cm^-1^ are characteristic of stretching vibrations of CH_3_, CH_2_, and CH groups. This is supported by a weak band at 1385 cm^-1^, which is indicative of bending vibrations of CH_3_, CH_2_, and CH groups. Medium-intensity bands at 1638 and 1617 cm^-1^ are indicative of the presence of a double bond within the molecule, possibly conjugated with a C=O carbonyl group. These bands are also characteristic of β-diketones in the phenolic form (-CO-C=C-OH). This observation is further supported by the stretching vibration bands of the OH group at 3554, 3477, and 3413 cm^-1^. Additionally, a broad band of weak intensity at 1148 cm^-1^ suggests the presence of a C-O-C group. The band at 619 cm^-1^ is characteristic of the bending vibrations of C-H bonds associated with double bonds.

## Discussion

Extreme microbial habitats are currently considered the most interesting for biotechnological research and are considered a rich source of new specialized metabolites [40-42].

The new actinomycete 312, isolated from soil samples of the arid region in the Almaty area exhibits pigment-producing capabilities and produces an antibiotic with a broad spectrum of activity. Microbial pigments are classified as secondary metabolites and are mainly produced due to metabolic disturbances under stressful conditions. Since the strain was isolated from an arid region, the pigments protect it from UV damage [43]. There is a close connection between pigmentation and the formation of secondary metabolites—when pigments are present, it is highly likely that antibiotics and other biologically active substances will also be produced.

The pigment produced by actinomycete strain 312 possesses antibiotic properties and is most effectively extracted with ethyl acetate, giving the extract a red color. It is extracted to a lesser extent with butanol, resulting in a dark purple color, and the least with acetone from the mycelium, which yields a faint purple extract. The antibiotic activity of antibiotic A-48-2 from the acetone mycelial extract is lower than that of the extracts from the culture broth.

Our findings are consistent with the literature: actinomycetes such as *Saccharomonospora azurea* [44] and *Streptomyces* spp. [45], *Streptomyces hygroscopicus* subsp. *Ossamyceticus* [46], and *Streptomyces torulosus* [47] produce blue, green, yellow, orange, purple, brown, and red pigments, which have therapeutic value [48]. Thus, a brown pigment synthesized by *Streptomyces* sp. strain BJZ10 was identified, known for its potential antimicrobial activity against gram-positive bacteria (the inhibition zone for *Bacillus cereus* was 14 mm) [49]. The actinomycete *Streptomyces hygroscopicus* subsp. *ossamyceticus*, isolated from the soil of the Thar Desert in Rajasthan, produces a yellow pigment with antibiotic activity [49]. The purified pigment exhibited a minimum inhibition zone of 15 mm against *Klebsiella* sp. and a maximum of 23 mm against vancomycin-resistant *S. aureus* in the disc diffusion method. An actinomycete strain was isolated from a leaf litter soil sample and identified as *Streptomyces* sp. The red pigment produced by strain JAR6 demonstrated strong antimicrobial activity against all test pathogens except *S. aureus*, *P. aeruginosa*, and *K. pneumoniae*. The bioactive compound responsible for the antimicrobial and anticancer activity of strain JAR6 was determined through spectroscopic analysis and is a metabolite of a red pigment [50].

Based on cultural and morphological characteristics, the genus and series of the strain were identified as *Streptomyces*, *Roseoviolaceus* series. Phylogenetic analysis of the 16S rRNA gene sequence of the actinomycete strain 312, compared with data from the international Greengenes database, showed that the strain belongs to the family *Streptomycetaceae*, genus *Streptomyces*, species *lateritius*. According to IR and UV spectral data, antibiotic A-312 lacks vibrations characteristic of naphthoquinones and has a maximum absorption in the visible region of the spectrum.

The antibiotic activity of the extracts was studied against laboratory gram-positive and gram-negative bacteria using the well diffusion method. The culture fluid and mycelium extracts showed high activity against gram-positive and gram-negative microorganisms.

It was found that during submerged fermentation of the producer, antibiotic A-312 accumulates both in the biomass (312-2) and in the culture broth (312-1). The antibiotic activity of 312-1 was studied against clinical strains of gram-positive (staphylococci, micrococci, streptococci) and gram-negative (*Klebsiella* and *Escherichia*) opportunistic pathogens with drug resistance. The clinical strains used as test microorganisms exhibited a high level of resistance to the main groups of medical antibiotics. The inhibition zone diameter for *S. aureus* 228 (MRSA) was 32 ± 0.2 mm, for *E. coli* 603 (ESBL) it was 20 ± 0.1 mm, for *Str. mitis* 683—23 ± 0.1 mm, and for *Mic*. spp. 132—28 ± 0.2 mm.

The data is in agreement with previous reports where *Streptomyces lateritius* Z1-26 illustrated broad-spectrum antibacterial activity against a broad range of fish pathogens. The *Streptomyces lateritius* Z1-26 was isolated from soil samples which showed broad-spectrum antibacterial activity against a broad range of fish pathogens [51].

A number of new granaticin type quinone antibiotics have been isolated from *Streptomyces lateritus* ATCC 19913. Spectroscopic evidence has been presented, leading to the structure elucidation of three new antibiotics, and the chemical relationship between members of the granaticin series has been studied. This research has resulted in the discovery of three novel antibiotics and clarified the chemical relationships among members of the granaticin series [52].

Using bioautography with *S. aureus* as the test organism, it was found that two components (component I and II) showed biological activity, with Rf values in the hexane-methanol:ethanol (3:1:1) system of 0.75 and 0.58 (antibiotic A-312-1), and in the n-butanol-acetic acid-water (4:1:5) system of 0.94 and 0.89, respectively. The absorption spectra of the complex antibiotic A-312 exhibited main maxima in the visible region at 565, 524, 491, and 409 nm.

It can be assumed that *Streptomyces lateritius* 312 strain synthesizes a new antibiotic and is significant as a producer of biologically active substances for the development of new pharmaceutical drugs.

## Figures and Tables

**Table 1:** Antagonistic properties of soil actinomycetes against gram-positive and gram-negative opportunistic bacteria (using the agar block method).

Isolate number	Diameter of the growth inhibition zone of the test microorganism, mm			
	*S. aureus*	*Sal. abony*	*E. coli*	*Kl. Pneumoniae*
1	2	3	4	5
25	20.3 ± 0.2	16.3 ± 0.5	0	15.1 ± 0.5
52	20.7 ± 0.5	12.4 ± 0.7	0	12.9 ± 0.2
61	20.1 ± 0.2	0	15.4 ± 0.7	13.4 ± 0.7
65	20.4 ± 0.1	0	0	0
71	25.2 ± 0.6	20.2 ± 0.2	18.4 ± 0.2	20.4 ± 0.3
73	25.3 ± 0.5	0	0	12.3 ± 0.5
78	25.7 ± 0.4	0	0	0
80	26.2 ± 0.3	12.5 ± 0.6	10.3 ± 0.7	0
86	25.1 ± 0.6	20.6 ± 0.8	17.3 ± 0.8	12.0 ± 0.4
119	27.8 ± 0.2	22.4 ± 0.6	11.5 ± 0.9	10.7 ± 0.2
124	27.3 ± 0.4	25.2 ± 0.2	0	13.2 ± 0.3
125	25.5 ± 0.1	20.2 ± 0.6	0	11.0 ± 0.1
135	27.6 ± 0.6	25.3 ± 0.5	0	15.4 ± 0.5
138	28.7 ± 0.5	23.1 ± 0.6	18 ± 0.6	20.1 ± 0.5
207	25.3 ± 0.2	19.9 ± 0.1	0	17.3 ± 0.1
208	30.5 ± 0.3	27.2 ± 0.5	21.2 ± 0.8	20.2 ± 0.2
211	30.1 ± 0.1	25.4 ± 0.1	22.2 ± 0.2	23.1 ± 0.6
225	25.9 ± 0.4	15.2 ± 0.3	0	18.0 ± 0.3
226	30.4 ± 0.2	22.4 ± 0.1	20.8 ± 0.5	18.7 ± 0.2
229	35.3 ± 0.4	25.3 ± 0.5	20.0 ± 0.2	20.0 ± 0.2
300	25.6 ± 0.5	18.0 ± 0.1	0	0
310	25.2 ± 0.1	20.9 ± 0.1	0	0
312	38.7 ± 0.4	30.2 ± 0.5	25 ± 0.8	28.2 ± 0.2
318	25.3 ± 0.3	19.4 ± 0.1	0	0
324	30.1 ± 0.3	25.2 ± 0.3	21.1 ± 0.1	20.0 ± 0.3
327	30.5 ± 0.1	27.8 ± 1.5	0	18.7 ± 0.2
330	32.8 ± 0.4	27.3 ± 0.5	20.0 ± 0.2	18.3 ± 0.5
333	30.3 ± 0.2	22.0 ± 0.1	18.5 ± 0.5	25.1 ± 0.3
342	33.2 ± 0.1	27.8 ± 1.5	22.4 ± 0.1	26.7 ± 0.2
350	28.3 ± 0.5	20.3 ± 0.5	0	0
358	30.4 ± 0.2	21.0 ± 0.3	0	0

**Table 2:** Cultural characteristics of *Streptomyces* 312 strain on diagnostic media.

Media	Growth	Color of aerial mycelium	Color of substrate mycelium	Pigment formation
Gauze’s mineral agar No. 1	Moderate	White to light-cream	Purple	Purple
Gauze’s organic agar No. 2	Good	White	Purple	Purple
Glucose-nitrate agar	Weak	-	Purple	Purple
Glucose-aspartic agar	Weak	-	Brown	Dusky pink
Sucrose-nitrate Czapek agar	Moderate	-	Purple	Purple
Glycerol-aspartic	Weak	Pink	Dark-brown	Burgundy-brown
Glycerol-nitrate agar	Weak	White	Purple	Purple
Sucrose-yeast Czapek agar	Good	White	Purple	Purple
Organic agar Prauser 79	Moderate	-	Purple	Purple
Starch-ammonia agar (ISP 4)	Moderate	White to pink	Pink to purple	Purple
Oatmeal agar (ISP 3)	Abundant	White and pink	Bluish to burgundy	Burgundy
Peptone-yeast agar with iron (ISP 6)	Good	White to pink	Dark-brown	Yellow to reddish-brown

**Table 3:** Evaluation of the antibiotic properties of strain 312 extracts using the well diffusion method.

Antibiotic extract	Diameter of growth inhibition zone (mm)			
	*S. aureus*	*S. abony*	*K. pneumoniae*	*E. coli*
1	45 ± 0.1	32 ± 0.3	26 ± 0.2	25 ± 0.2
2	35 ± 0.3	28 ± 0.2	22 ± 0.3	20 ± 0.2
3	32 ± 0.2	22 ± 0.2	18 ± 0.3	17 ± 0.3

Extracts from the culture liquid: 1—ethyl acetate, 2—butanol; extracts from the mycelium: 3—acetone.

**Table 4:** Drug resistance of clinical strains of opportunistic pathogens with multiple drug resistance.

Clinical Strains	Antibiotic Resistance Level										
	1	2	3	4	5	6	7	8	9	10	11
1	2	3	4	5	6	7	8	9	10	11	12
*S. aureus* 228	R	S	S	S	S	S	S	S	S	S	S
*S. haemolyticus* 878	R	R	S	S	R	S	S	S	S	S	R
*S. epidermidis* 948	R	R	R	I	S	R	S	S	R	R	R
*Str. mitis* 683	S	S	R	R	R	R	S	S	R	R	S
*Micrococcus* spp. 132	R	R	S	S	R	I	S	S	S	S	R
*K. pneumoniae* 948	R	R	R	R	S	S	R	S	S	S	I
*K. pneumonia* 842	R	R	R	R	R	R	-	R	S	R	R
*E. coli* 603	R	R	R	I	S	S	S	S	S	S	S

Antibiotics for gram-positive bacteria: 1—benzylpenicillin, 2—oxacillin, 3—lincomycin, 4—gentamicin, 5—erythromycin, 6— tetracycline, 7—vancomycin, 8—rifampicin, 9—levofloxacin, 10—ciprofloxacin, 11—cefazolin. Antibiotics for gram-negative bacteria: 1—benzylpenicillin, 2—ampicillin, 3—cefazolin, 4—cefepime, 5—cefoxitin, 6—imipenem, 7—doxycycline, 8— gentamicin, 9—amikacin, 10—levofloxacin, 11—ciprofloxacin. R—resistant; I—intermediate; S—sensitive.

**Table 5:** Activity of natural antibiotic A-312 against clinical strains with multiple drug resistance.

Antibiotic 312	Activity of natural antibiotic 312 against clinical strains with multiple drug resistance (inhibition zones, mm)								
	*K. pneumonia*е 948	*K. pneumoniae* 842	*E. coli* 603	*E. coli* 446	*S. aureus* 228	*S. haemolyticus* 878	*S. epider*. 948	*Str. mitis* 683	*Micrococcus* spp. 132
312-1	20 ± 0.2	17 ± 0.1	20 ± 0.1	15 ± 0.3	32 ± 0.1	20 ± 0.3	18 ± 0.1	20 ± 0.1	20 ± 0.2
312-2	15 ± 0.1	12 ± 0.3	15 ± 0.1	10 ± 0.1	25 ± 0.2	15 ± 0.2	12 ± 0.2	12 ± 0.3	13 ± 0.1

**Table 6:** Chromatographic behavior of the components of antibiotic A-312.

Solvents	Antibiotic 312-1		Antibiotic 312-2
	Rf components (A)	Rf components (B)	Rf components
n-butanol-acetic acid-water (4:1:5)	I-0.94; II-0.89	I-0.94; II-0.89; III-0.54	I-0.94; II-0.89
n-butanol-acetic acid-ethanol (1:1:4)	I-0.95; II-0.94	I-0.96; II-0.92; III-0.65	I-0.97; II-0.95
n-butanol-ethanol-water (1:4:1)	I-0.97; II-0.95; III-0.91	I-0.97; II-0.90; III-0.87; IV-0.83	I-0.95; II-0.90
hexane-methanol-chloroform (3:1:2)	I-0.75; II-0.68; III-0.59; IV-0.46; V-0.1	I-0.75; II-0.68; III-0.59	I-0.75; II-0.42; III-0.35
chloroform-methanol (7:1)	I-0.98; II-0.95	I-0.98	I-0.95; II-0.81
hexane-methanol-ethanol (3:1:1)	I-0.75; II-0.58; III-0.4	I-0.75; II-0.58; III-0.4	I-0.75; II-0.58

(A) From ethyl acetate extract. (B) From butanol extract.
